# Transactions of the Chicago Society of Physicians and Surgeons

**Published:** 1874-06-01

**Authors:** Plym. S. Hayes


### TRANSACTIONS OF THE CHICAGO SOCIETY OF PHYSICIANS AND SURGEONS.

### ANNUAL MEETING OF MAY 11TH, 1874.

*Reported by Plym. S. Hayes, M. D.*

THE Society met in the parlor of the Grand Pacific Hotel, the President in the Chair.

The Secretary read the minutes of the two preceding meetings.

Drs. G. H. Chapman and Wm. Rofe were then elected to membership.

The Secretary read the annual reports of the Treasurer and Secretary, which, on motion of Dr. Emmons, were accepted. It was also voted to present the report of the Secretary for publication in the *Chicago Medical Journal*, and the MEDICAL EXAMINER.

Subjoined is the Report:

SECOND ANNUAL REPORT OF THE SECRETARY OF THE CHICAGO SOCIETY OF PHYSICIANS AND SURGEONS FOR THE YEAR ENDING, MAY 11TH, 1874.

*Mr. President and Gentlemen* :

In the presentation of the Report of the Secretary for the year, which closes with this date, I have thought it proper to prepare an Abstract of the Proceedings of the Society for the period covered by the Report. A similar Abstract was read to you at the last annual meeting, and at the close of the year 1873, I published an Abstract of Proceedings for that period also. The reasons for this step were based upon the fact, that a large number of medical men habitually renew their subscriptions for medical periodicals at that season, and consider it a fitting occasion for subscribing for new ones. I therefore deemed it advisable to present our claims upon their consideration at that time, by supplying them with information as to the work accomplished during the year then brought to a close. I refer to this now, merely to explain that the subjoined Abstract covers a period from May 12th, r873, to May 11th, 1874; and differs from the last in the *inclusion* of Reports from January to May of this year, while those proceedings which extended from January to May of 1873, are purposely omitted:

I.—REPORTS.

1. Annual Reports of Officers for year 1872-3.
2. Annual Report of Surgical Section, (3 papers): * (*a*) On Uranoplasty and Staphylorrhaphy; * (*b*) on Stricture of the Urethra and Rectum; (*c*) on the Electro Therapeutics of Surgery.
3. Annual Report of the Section on Pathology, (2 papers): (*a*) On General Pathology; (*b*) on Pathology of the Nervous System.
4. Reports of the two special committees on Cholera: * (*a*) On the treatment of the Epidemic of 1873; † (*b*) on the Pathology of the Disease. The paper being illustrated by sections of the human intestines, magnified by the solar microscope, and illuminated by the oxy-calcium light.
5. Report on the Endemic in Chicago with map of localities.

*6. Annual Report of Section on Gynecology for 1873-4.

7. Report on the Therapeutic Value of Cosmoline.

II.—PAPERS.

1. On the Progress of Medicine.
2. On some Questions in Therapeutics.

*3. On the Faradaic Currents.

*4. On Waxy Kidney.

*5. On A Review of Hall's Surgery, London, 1565.

*6. On the Physiological Relations of Alcohol.

*7. On Lime Vapor in Membranous Croup.

*8. On the Cervix Uteri, Before, During and After Labor.

†*9. On the Marsh Fungi Productive of Malarial Disease—Illustrated by colored sketches of Palmellae, microscopical specimens of sporules, blood, saliva, &c.

*10. Abstracts of the Literature of the Cholera of Later Days.

III.—REPORTS OF CASES.

1. False Pregnancy.
2. Extirpation of Testis.

*3. Chronic Inflammation of Stomach with magnified and illuminated sections—exhibition of stomach in gross. Report of Microscopy and Pathology.

4. Cases of Cholera in Chicago, 1873.
5. Cases of Cholera in Louisville, 1856.
6. Additional Cases in Chicago, 1873.
7. Additional cases in Chicago, 1873.

*8. Thirteen Cases of Uterine Fibroid Tumors, treated with hypodermic injections of Ergotine.

9. Foreign Body in the Urethra.
10. Death from Gunshot Wound of the Cerebral Sinuses.
11. Sciatica Resulting from Suppressio Mensium relieved by the thermo-electric bath.

*12. Two Cases of Parietal Gaseous Abscess of the Abdomen.

*13. Empyema and Paracentesis.

*14. Re-amputation of Leg — (Esmarch's method).

15. Three Cases of Operation for necrosis of femur—(Esmarch's method).
16. Amputation of Fingers — (Esmarch's method).
17. Accidental Amputation at Shoulder-joint.

*18. Delirium tremens.

19. Fracture of Pelvis.
20. Pott's Disease.
21. Stricture of Urethra.
22. Procidentia of Bowel mistaken for Umbilical cord in Parturition, with fatal results.
23. Aspiration of an Ovarian Cyst.
24. Suicide by Opium Poisoning.

*25. Abstract of Forty-nine cases treated in the Woman's Hospital of Illinois.

*26. Case of Ovariotomy for colloid degeneration.

*27. Case of Operation for Staphylorraphy, the anæsthesia being produced by the atomizer.

IV.—SPECIMENS PRESENTED.

1. Ossified Cardiac Valves.
2. Cancer of the Uterus, with report of case.
3. Hypertrophied Heart, with report of case.
4. Uterus in case of suspected abortion.

*5. Calcareous degeneration of pericardium and greatly enlarged ureter, with report of case and microscopy.

6. Cholera dejections under the microscope, with history of the case.
7. Patient with complete loss of epiglottis and unimpaired power of deglutition.

Number of meetings during the year: 23

regular and 1 special. Number of new members added during year, 43. Total attendance during the year, 418. Average attendance each meeting, 18.2.

The following is a brief résumé of the special action taken by the Society during the year: The Sections on Medical Science have been increased in number; rules regulating the invitations to non-members, for the purpose of participating in debate, have been passed; a precedent has been established by vote, requiring the Censors to report in the case of every candidate for membership, either favorably or unfavorably, doing away with the neglect to report on names of suspected individuals; a resolution has prevailed, urging the attention of our Congressional Representatives to the Rule for Regulating the Rank of Medical Officers of the Army, and a By-Law has been added authorizing the annual appointment of a Committee on Necrology. Women have been expressly excluded from membership by vote. The Committee on Clinical Reports—newly created—has added largely to the character and interest of the Society's transactions. Delegates have been chosen for the representation of this organization to the State Medical Society, and a committee of your appointment is now cooperating with a similar committee from the Chicago Medical Society, for the purpose of giving an entertainment to the members of that Society, who are expected to be our guests during the coming week. It is proper to state here, also, that a nucleus for a Medical Library has been found in the three volumes of the "Catalogue of the Library of the Surgeon General's office," presented to the Society by the Asst. Surgeon General of the Army. Lastly, the change in the locality where our meetings have been held is one which was effected only with hesitancy, and after considerable deliberation. It is believed that at present there are none who doubt the wisdom of such action when the exigency for it occurred. Certainly, in point of attendance and interest, there has been an impovement which is commensurate with the value of the Society's transactions.

I desire to bring to your attention, before closing this Report, certain suggestions whose value lies chiefly in the fact that they arise from a practical acquaintance with the work of this Society during the year just closed, and I recommend such action in the future as will embody the views of members upon the points at issue.

And, first, it seems to me to be desirable to establish a regulation which shall prohibit the admission of medical men to our ranks until they are graduates of at least *one* year's standing. It seems right and proper that a period of probation should be fixed before the expiration of which, the alumni of our Medical Schools cannot be candidates for membership. For, in the first place, many of them are anxious to avail themselves of the full privileges of the doctorate, before they are really settled in life or in practice; and, secondly, such a regulation is to be found in most, or many, of the rules of associations similar to our own.

Again: The Code of Ethics of the American Medical Association—a code which, admirable as it is in all respects, is a part of the regulations

---

*Note to Abstract.*—Papers marked † received a vote of thanks from the Society.

Papers marked * have been published in the medical periodicals.

of the Society—provides that medical men who carry on the business of a druggist, or are interested, financially, in the traffic of a drug-store, are debarred from the full privileges of the honorable profession of which we are members. This is a subject upon which it is difficult to speak. On several occasions during the past year, statements have been made to me to the effect that individuals who were members of this Society were interested in the prosecution of the business of a druggist. I do not know if these statements are founded upon fact, but if so, certainly they demand the earnest and immediate attention of this Society. Not that these individuals, if such there be, have been guilty of any offence which reflects upon their character as upright or moral members of the social community in which they live. Far from it. The question is simply this: "Shall those who rely exclusively upon the rewards of professional labor, and such sources of private income as they may possess, for the support of themselves and their families, admit to all the privileges, safeguards and honors of the profession, those who appeal to the public for patronage of another sort?" The question has been so decidedly answered in the negative, by the voice of medical practitioners throughout our country and abroad, that I shall not discuss it here. I merely present it to you for the answer which I am confident you will not fail to return. A third suggestion I consider of so much practical moment that I wish I had the time to dwell upon its details. It seems to me desirable to arrange, and place in the hands of every member, an "order of papers, reports and cases," for certain dates of the *entire* year. This order, which should be, in the parliamentary sense, a "special order," might be printed on slips, and distributed for file by every interested individual. It should state:

1st. The dates upon which the *sections* are to report. Formerly the sections were made so large that they included most of the members of the Society. With our present numbers, this is obviously impracticable, and it would seem desirable to constitute the sections, as contemplated originally in the resolution providing for them, in such a manner that three members only shall be assigned to each section. These members should be selected for their special fitness or willingness to prepare reports on the subjects contemplated, and to pledge themselves to present such reports on certain fixed dates, in order that the dates assigned for other business should not be encroached upon. The plan of these sections has attracted some attention from our friends who are not connected with this organization. It is the result, doubtless, of the publication of the annual reports in the medical journals of our city. I was recently waited upon by a delegation from a sister Society, who requested information respecting the ordering of these sections with us, and I was pleased to give the fullest account of the method—a method which they have since seen fit to adopt. These reports generally present an abstract of material, published during the year, upon the subject assigned to each particular sub-section. But it is desirable to remember that *original* contributions are not excluded from these reports. They are, by regulation, required to present "new or interesting" facts, and the assignment of the sections to members having special interest in the field of medicine, or surgery allotted to them, would seem to indicate that "new or interesting" observations of an *original* character might be also profitably reported.

2d. The second class of business which should be calendered in the "special order for the year," is, the reports of the Clinical Committee. As I have already referred to their work, I need only say here that it promises to add greatly to the interesting features of our meetings during the coming year, and it would seem proper that the present chairman, whose appointment now dates back but a few weeks, should be continued in a position which is one of responsibility, and which he filled eminently to the satisfaction of his associates in the various hospitals represented in the Society.

And, lastly, the "special order for the year" should include the names of these members who are willing to pledge themselves to present papers at certain dates in the year, (to be specially designated,) together with the subjects upon which they propose to write. By this means, every individual will have some knowledge of the work contemplated by the Society for the year, and can prepare himself in advance, to take part in the discussions which should follow, with credit to himself and with advantage to others. I will not repeat here the remarks quoted by me in the First Annual Report, which I had the honor to submit to this Society in May, 1873. They were from the lips of the distinguished President of the New York Academy of Medicine. They set forth with great plainness and emphasis, the well-known fact that only those, who from long experience as writers or teachers were qualified to speak extempore upon medical subjects, could do so, without previous study and investigation, in a manner at all satisfactory to themselves or others. Preparation—careful, systematic preparation, on the topics to be brought forward for discussion—could only make those discussions valuable to the scientific world. The notices of meetings, which are addressed semi-monthly to the members of the Society, can rarely, for obvious reasons, present fully the subjects to be discussed, and a reference to the "special order of the year,"—if printed and distributed as recommended above—would at once indicate, on many occasions, exactly what business was to be brought forward at any given date.

My apology for thus presuming to indicate, what seem to me to be suggestions of a practical character deserving your attention, and for setting them before you somewhat at length, must be found in the interest which is natural to one who has been actively engaged in executing the work of the Society since its organization. And I cannot conclude without expressing my thanks in general to all those who have efficiently contributed to assist in the pleasant duties which have devolved upon me. To Dr. P. S. Hayes, the Reporter of the Society during the last year, I am under obligations for valuable aid in recording and reporting our transactions. To the Editors of the two medical journals, the Society is deeply indebted, for the publication of all such reports in full, as have been forwarded to them; and their action, in my opinion, has contributed more to the respect entertained for the Association by the profession, in this and other cities of our country, than any other agency. Not infrequently have I noted quotations in Eastern periodicals, of later dates, especially, from the pages to which they have had access in their exchanges, which have given clinical and other observations, first brought before the medical world in the meetings of this Society.

Nor can I conclude without an expression of my congratulations upon, not merely that which you have already accomplished, but that which you are sure to accomplish in the future.

On the 14th day of August, 1843, there was erected in the City of Bourg, in France, a bronze statue, representing a man in the attitude of meditation. One hand was placed upon the heart of a child standing by his side, and it seemed, by its touch, to take note of the pulsations in that region. At the feet of the two figures reclined the image of an inanimate body, beside which a symbolic lamp was burning, as if to illuminate the sombre domains of Death.

This monument, the work of the great French artist, David, was designed to commemorate the life and work of the immortal Bichat, whose physiological researches seemed to have opened to him the portals of the secret chambers of Life and Death. The problems before us are those which were before him—the problems of life and death—problems, whose solution has, for centuries, taxed the greatest intellects of our profession—problems, whose gravity and tremendous importance impress us with the need of sober, steady and resolute exertion. It is ours to keep alight the sacred flame represented in the sculptured figure of the artist, the lamp fed by the accumulated oil of generations of our co-laborers in science. It is ours to perpetuate such a light as shall illuminate and, at the same time, involve no danger in the fervency of its blaze. The Esquimaux is said to trim his seal-oil lamp in his hut of snow, fearful lest the icy walls should melt before its presence. Let *us* build of such material, that the reflection of light upon the walls which we rear may only serve to render them the more beautiful and enduring.

Chicago, May 11, 1874.

In compliance with a motion, the President named Drs. Trimble, F. H. Davis, and Blake, a Committee on Nomination of Officers. The committee withdrew, and after consultation presented the following names as candidates for the various offices: For President, Dr. John Bartlett; for Vice-President, Dr. John E. Owens; for Secretary and Treasurer, Dr. J. Nevins Hyde; for Censors, Drs. D. B. Trimble, J. H. Hollister, and Walter Hay; for Reporter for the medical press, Dr. Ralph E. Starkweather.

On motion of Dr. Andrews, the Secretary was instructed to cast a unanimous ballot for the nominees.

Dr. Bartlett, President-elect, was then conducted to the chair by Drs. Trimble and Owens.

Dr. Delafontaine then delivered a lecture on the examination of blood, by means of the spectroscope. He thought the spectroscopic examination of old blood was more definite and conclusive in its results than that by the microscope. After the lecture he showed the absorption spectra of old and fresh blood.

A vote of thanks was tendered to Dr. Delafontaine for the communication of the result of his researches to the Society.

Dr. E. Andrews, one of the Committee on Hospital Reports, then read a paper on the mortality of amputations in the Western, as compared with those in the Eastern States, and Europe. From statistics that he had gathered and compared, he found that in four of the major amputations, viz.: of the thigh, leg, arm, and fore-arm, the mortality in the States surrounding the lakes was nearly the same, in hospital and civil practice, whether in the city or country. The mortality in the Western was less than in the Eastern States; and that of this country, collectively, less than that of England; while a less number died in England from these amputations than in France, where the mortality was the greatest. He dwelt on the conveying of septicemic poison in the form of dust, and on ventilation, and the cleansing of beds.

The following table shows the mortality after amputations of the arm, fore-arm, thigh and leg, in different countries, (excluding amputations through the joints):

| MORTALITY. | *Per ct.* |
|---|---|
| Hospitals of Chicago | 23 |
| Private practice in the Cities and States around Lake Michigan | 22 |
| Country practice in the same States | 20 |
| Hospitals of the Atlantic States | 26 |
| Hospitals of Great Britain | 42 |
| Hospitals of Paris | 62 |

Dr. Trimble moved that a vote of thanks be extended to the retiring officers, and the motion prevailed.

The following motion of Dr. Simon was adopted.

*Resolved*, That the delegates to the State Medical Society, from this Society, recommend to that body the advisability of memorializing Congress on the subject of the remission of duties on scientific books.

After a discussion of certain parliamentary usuages the Society adjourned.

